# Disparity between dorsal and ventral networks in patients with obsessive-compulsive disorder: evidence revealed by graph theoretical analysis based on cortical thickness from MRI

**DOI:** 10.3389/fnhum.2013.00302

**Published:** 2013-07-03

**Authors:** Seung-Goo Kim, Wi Hoon Jung, Sung Nyun Kim, Joon Hwan Jang, Jun Soo Kwon

**Affiliations:** ^1^Department of Brain and Cognitive Sciences, Seoul National UniversitySeoul, South Korea; ^2^Research Group for Cortical Networks and Cognitive Functions, Max Planck Institute for Human Cognitive and Brain SciencesLeipzig, Germany; ^3^Clinical Cognitive Neuroscience Center, SNU-MRC, Seoul National University HospitalSeoul, South Korea; ^4^Department of Psychiatry, College of Medicine, Seoul National UniversitySeoul, South Korea

**Keywords:** obsessive-compulsive disorder, magnetic resonance imaging, cortical thickness, structural connectivity, graph theoretical analysis, network efficiency, small-worldness, dorsal-ventral imbalance

## Abstract

As one of the most widely accepted neuroanatomical models on obsessive-compulsive disorder (OCD), it has been hypothesized that imbalance between an excitatory direct (ventral) pathway and an inhibitory indirect (dorsal) pathway in cortico-striato-thalamic circuit underlies the emergence of OCD. Here we examine the structural network in drug-free patients with OCD in terms of graph theoretical measures for the first time. We used a measure called efficiency which quantifies how a node transfers information efficiently. To construct brain networks, cortical thickness was automatically estimated using T1-weighted magnetic resonance imaging. We found that the network of the OCD patients was as efficient as that of healthy controls so that the both networks were in the small-world regime. More importantly, however, disparity between the dorsal and the ventral networks in the OCD patients was found in terms of graph theoretical measures, suggesting a positive evidence to the imbalance theory on the underlying pathophysiology of OCD.

## 1. Introduction

Obsessive-compulsive disorder (OCD) is an anxiety disorder characterized by intrusive, distressing thoughts and ritualistic, repetitive behaviors (American Psychiatric Association, 1994). The most widely accepted neuroanatomical model of OCD has suggested the involvement of a direct and an indirect cortico-striato-thalamic (CST) pathway (Cummings, 1993; Saxena et al., 1998). In this model, the direct pathway involves in an excitatory input to the internal part of globus pallidus that leads to a disinhibition of thalamus and increased excitation of prefrontal cortex, whereas the indirect pathway involves in an inhibitory input to the external part of globus pallidus that evokes an increased inhibition of thalamus and decreased excitation of prefrontal cortex (Mataix-Cols and van den Heuvel, 2006). Although the oversimplification of the model is questioned (Menzies et al., 2008; Milad and Rauch, 2011), the dichroism has been a well-known basis in approaching the disorder for a long time (Saxena et al., 1998).

Functional studies driven by the CST model have converged on altered activation in patients with OCD in relation to healthy controls in basal ganglia, caudate nucleus, thalamus, orbital frontal cortex, cingulate gyrus, dorsal lateral cortex and parietal regions, using single-photon emission computed tomography (SPECT), positron emission tomography (PET) or functional magnetic resonance imaging (fMRI) (Whiteside et al., 2004; Friedlander and Desrocher, 2006). Despite of the inconsistencies in the literature to some degree, many papers reported higher activations from OCD patients in orbital frontal cortex and basal ganglia, in particular striatum, which have been related to the “hyperactivation of ventral fronto-strital system”. In addition, lower activations from OCD patients in dorsal lateral prefrontal cortex and anterior cingulate gyrus, which have been known as the “hypoactivation of dorsal fronto-strital system” (Saxena et al., 1998; Remijnse et al., 2005; Oh et al., 2012; Mataix-Cols and van den Heuvel, 2006).

In relation to the functional findings supporting the CST hypothesis, structural neuroimaging evidences of the abnormalities in OCD patients have been cumulated. Structural alterations were mainly localized in prefrontal regions and basal ganglia. In meta-analyses, gray matter densities in bilateral anterior putamina were found to be higher in OCD patients than healthy controls, and those in dorsal prefrontal regions were found to be lower in OCD patients (Radua and Mataix-Cols, 2009; Rotge et al., 2010). In addition, diffusion tensor imaging (DTI) studies have shown that smaller fractional anisotropy (FA), which has been commonly used to characterize local diffusion and thus to infer white matter integrity (Basser, 1995), were found in clinical population with OCD in the anterior part of cingulum, corpus callosum and other white matter regions in frontal and parietal lobes (Szeszko et al., 2005; Garibotto et al., 2009; Ha et al., 2009; Bora et al., 2011; Koch et al., 2012; Nakamae et al., 2011; Oh et al., 2012).

It should be noted that the previous structural studies mentioned above have mainly focused on differences in local morphology using massive univariate frameworks such as voxel-based morphometry (VBM; Ashburner and Friston, 2000) or tract-based spatial statistics (TBSS; Smith et al., 2006). Due to its complex nature of brain network, local alterations might not be sufficient to understand the disorder. As needs for investigating connectivities within the CST circuits and between other brain regions have been motivated in the previous literature on OCD (Remijnse et al., 2005; Mataix-Cols and van den Heuvel, 2006; Menzies et al., 2008), a seed-based correlation method (Harrison et al., 2009; Jang et al., 2010) and a whole-brain graph analysis have been used in functional studies (Zhang et al., 2011).

While it is demonstrated that network analyses are capable of investigating the properties of human brains that could not be described using the conventional analyses (Bullmore and Sporns, 2009), relatively new neuroimaging modalities such as DTI and resting-fMRI have been mainly used for the brain connectivity studies. In order to exploit conventional anatomical scans such as T1-weighted MRI, a network analysis based on the correlation of local morphology has been proposed as an alternative framework to examine structural networks in human brains (Worsley et al., 2004; Lerch and Evans, 2005; He et al., 2007; Bernhardt et al., 2011).

In the studies, gray matter is characterized by cortical thickness in a sub-millimeter resolution using cortical surface reconstruction techniques (Dale et al., 1999; Fischl and Dale, 2000; MacDonald et al., 2000). This approach, which only requires T1-weighted MRI, assumes that the positive correlation of cortical thickness may reflect the anatomical connectivity, presumably because of common experiences, shared trophic or maturational influences (Lerch et al., 2008; He et al., 2009; Raznahan et al., 2011). These networks constructed based on cortical thickness have shown their resemblance to DTI-based networks (Gong et al., 2012) and similar modular structures with known functional modules (Chen et al., 2008, 2011). More recently, the cortical thickness-based network in developing brains notably overlapped a functional network known as default-mode-network (DMN; Raznahan et al., 2011).

Here we apply the cortical thickness network analysis on patients with OCD. To our best knowledge, there has been no preceding study to examine graph theoretical measures of brain networks in patients with OCD based on the correlation of cortical thickness so far. As the cortical thickness network analysis has shown its ability to detect reliable and meaningful attributes of human brains in healthy population (He et al., 2007; Chen et al., 2008, 2011; Gong et al., 2009, 2012) and clinical populations with disorders such as multiple sclerosis (He et al., 2009), Alzheimer's disease (He et al., 2008) or temporal lobe epilepsy (Bernhardt et al., 2011), we expect to find alterations in the brain of patients with OCD in terms of network properties, in particular, with a supporting evidence for the dorsal-ventral imbalance in the CST circuits (Saxena et al., 1998; Mataix-Cols and van den Heuvel, 2006), as well as abnormalities in other circuits including dorsal anterior cingulate cortex (Milad and Rauch, 2011) and parietal cortex (Menzies et al., 2008).

Our main contributions include: (1) performing a cortical thickness network analysis on drug-free patients with OCD, (2) investigating graph theoretical measures in the perspective of the major hypothesis of OCD at a network-level (Latora and Marchiori, 2001) and a node-level (Achard and Bullmore, 2007), and finally (3) examining the pathophysiology of OCD in terms of disparity between dorsal and ventral networks, as recently shown as a spatial bias in FA alteration within corpus callosum in OCD patients (Oh et al., 2012).

## 2. Materials and methods

### 2.1 Participants

We recruited 32 patients who fulfilled the criteria for OCD in DSM-IV (American Psychiatric Association, 1994) *via* the OCD clinic at Seoul National University Hospital (Seoul, Korea). The patients were diagnosed using the Structured Clinical Interview for DSM-IV (SCID; First et al., 1996). All of the patients with OCD were drug-free: 23 patients were drug-naïve, and the other 9 patients were unmedicated for at least 4 weeks at the time of inclusion. Four patients were assessed to have personality disorders in addition to OCD: three were with obsessive-compulsive personality disorders and one was with schizotypal personality disorder. In addition to the patients, we also recruited 35 age- and gender-matched controls (HC) using the SCID Non-patient Version to confirm that none of the controls was with Axis I psychiatric disorders. The exclusion criteria for both patients and control included lifetime history of psychosis, bipolar disorder, major depressive disorder, substance abuse or dependence, significant head injury, seizure disorder or mental retardation. All participants were right-handed. The severity of depression and anxiety was measured by self-reporting Beck's Depression Inventory (BDI; Beck et al., 1961) and Beck's Anxiety Inventory (BAI; Beck et al., 1988), respectively. The severity of OC symptoms was assessed with clinician-administered Yale-Brown Obsessive-Compulsive Scale (Y-BOCS; Goodman et al., 1989). The institutional review board (IRB) of Seoul National University Hospital (H-1209-025-424) approved the present study. All participants were fully instructed about the procedures of scanning and assessment and then submitted written informed consents.

### 2.2. Image acquisition and graph construction

We obtained magnetic resonance imaging (MRI) using 1.5T MAGNETOM Avanto syngo scanner (Siemens, Erlangen, Germany). T1-weighted 3D images were acquired with the following parameters: TR = 1160 ms, TE = 4.76 ms, FOV = 230 mm, flip angle = 15°, voxel size: 0.45 × 0.45 × 0.90 mm, volume dimension: 350 × 263 × 350 mm.

**Figure 1:**
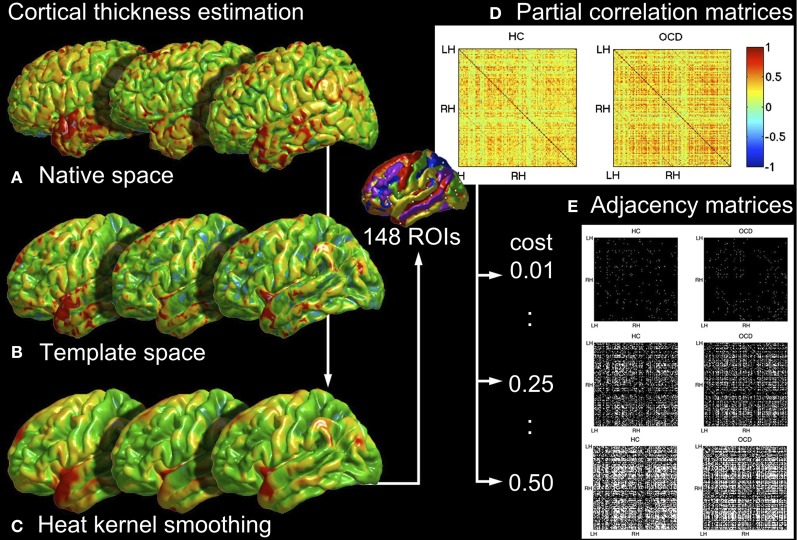
**The illustration of analysis steps to construct networks from MRI.** The cortical thicknesses are estimated in native spaces (**A**, section 2.2.1), normalized into the template space (**B**, section 2.2.2), and smoothed by a heat kernel based on Laplace-Beltrami eigenfunctions (**C**, section 2.2.3). Partial correlations are computed over 148 ROIs (**D**, section 2.2.4) and binarized to obtain adjacency matrices at a certain rewiring cost (**E**, section 2.2.5).

The steps of image analysis are illustrated in Figure 1. To compare brain networks between the patients and the controls at the final stage of analysis, we estimated cortical thicknesses from MRIs and constructed brain networks based on them. The detailed steps of the present analysis are explained in the followings. The analysis was carried by custom MATLAB (Mathworks Inc., Natick, MA, USA) codes, if not otherwise specified.

#### 2.2.1. Cortical thickness estimation

The reconstruction of cortical surfaces and the estimation of cortical thickness were performed using FreeSurfer^1^. As in its standard pipeline (Dale et al., 1999), the intensity of T1-weighted images were normalized and the bias of B0 field was corrected. Then the images were resampled in a unit millimeter isovoxel. An inner cortical surface (the interface between white matter and gray matter) and an outer cortical surface (the interface between gray matter and cerebrospinal fluid) were modeled as triangular tessellation. The cortical thickness was computed by averaging distances from the inner surface to outer surface and the distance from the outer surface to the inner surface (Fischl and Dale, 2000).

#### 2.2.2. Spatial normalization and resampling on a template surface

The estimated cortical surfaces were spatially normalized onto a given template surface, called *“fsaverage6”* with 40962 vertices for each hemisphere, using curvature matching technique to align major sulci patterns (Fischl et al., 1999). Then the cortical thickness was resampled onto the template surface, resulting in the correspondence of measures across all participants. This normalization enables a direct comparison of a vertex or a set of vertices across participants.

#### 2.2.3. Heat kernel smoothing *via* laplace-beltrami eigenfunction

Individual cortical thickness maps on the template surface were smoothed using a heat kernel smoothing technique based on Laplace-Beltrami (LB) eigenfunctions (Seo et al., 2010; Kim et al., 2011b; Seo and Chung, 2011). The surface-based smoothing reduces the impact of possible abrupt noise or errors from MRI scanning, surface reconstruction and thickness estimation, thus increases statistical power (Chung et al., 2005; Lerch and Evans, 2005). In addition, due to its analytic formulation, the heat kernel smoothing *via* LB eigenfunctions has a benefit of circumventing numerical errors in conventional smoothing techniques based on iterations. Theoretical details are explained in somewhere else (Seo et al., 2010). In this paper, we used 4000 orthonormal bases of LB eigenfunctions. The measurements were smoothly recovered with the bandwidth parameter σ of 10 mm, using freely available MATLAB codes by Moo K. Chung^2^.

#### 2.2.4. Partial correlation between ROIs

Automatic parcellations of gray matter into 74 regions-of-interest (ROIs) per hemisphere were adapted from (Fischl et al., 2004; Destrieux et al., 2010), which was included in FreeSurfer as *“Destrieux 2009 atlas”*. Although the 148 ROIs are less uniform in terms of area (mean area = 13.73 ± 9.68 cm^2^) than in a high-resolution parcellation with about 1000 ROIs (mean area ~1.5 cm^2^ with standard deviation less than 0.15 cm^2^) used in Hagmann et al. (2007, 2008), the anatomical significance of the current parcellations assists us in interpreting results while reducing the computational loads in permutation tests as described in the section 2.4. Thickness measures were averaged in each ROI and used in further analysis.

We computed partial correlation between the ROIs while factoring out the effect of age and gender, as well as the mean of measures, as previous cortical thickness network studies (He et al., 2007, 2008, 2009; Bernhardt et al., 2011). First we fit such a general linear model (GLM) as

(1) $$c(x)=\beta_{0}+\beta_{1}a+\beta_{2}g+\varepsilon ,$$

where **c**(*x*) is the vector of the cortical thickness of the *x*-th ROI for individual participants, β_*k* = 0, 1, 2_ are unknown parameters to estimate, **a** is the vector of ages, **g** is the vector of genders and ϵ is the vector of Gaussian random noise. Once we estimated the parameters with the least square method, the residuals $c(x)-\hat{c}(x)$ of the GLM were used to compute a correlation matrix **R** = [*r*_*xy*_] ∈ ℝ^148 × 148^ as

(2) $$r_{xy}=\text{corr}(c(x)-\hat{c}(x),c(y)-\hat{c}(y)),$$

*corr*(**i**, **j**) is the Pearson product of two vectors **i** and **j** as

(3) $$\text{corr}(i,j)=\frac{\sum ij-\left[\left(\sum i\sum j\right)\text{​}/n\right]}{\left[i^{2}-{\left(\sum i\right)}^{2}/n\right]\left[j^{2}-{\left(\sum j\right)}^{2}/n\right]},$$

where *n* is the number of the elements of **i** or **j**. Since we are interested in the anatomical connectivity due to neuronal associations under the same assumptions in the previous network studies based on cortical thickness (He et al., 2007, 2009; Bernhardt et al., 2011), we do not examine anti-correlations in this paper. Although negative association should also be studied in the future to reveal the biological mechanism of the cortical thickness interdependence (Lerch et al., 2008; Raznahan et al., 2011), it would be beyond the scope of the current study.

#### 2.2.5. Network construction for different rewiring costs

For it is known that the *rewiring costs*, or the density, of a network critically affects the graph theoretical properties and topological characteristics of network (Eguíluz et al., 2003; Latora and Marchiori, 2003; Achard et al., 2006; Gong et al., 2009), we controlled the costs to compare the brain networks of the OCD patients with that of the controls. When an undirected and unweighted graph 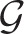 is written as a set of two sets as


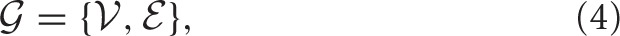


where 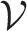 is the set of vertices and ℰ is the set of edges, then the cost of a graph 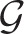 is given as


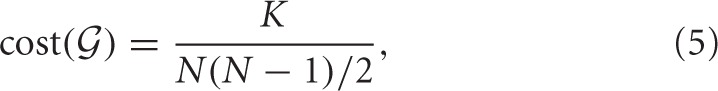


where *K* = |ℰ| as the number of edges and 
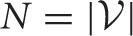
 as the number of vertices in the graph 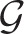. Note that *N*(*N* − 1)/2 is the largest number of possible *K*. Thus the cost equals to zero when there is no connections and the cost equals to one when every node is directly connected to all the other nodes. We binarized the correlation matrices so that they have the equivalent cost, ranging from 0.01 to 0.50 with a step of 0.01. It resulted in 100 (50 costs × 2 groups) adjacency matrices **A**_*g*, *c*_ with the dimensionality of 148 × 148, where *g* is the group index (*g* = 1 for the controls; *g* = 2 for the OCD patients) and *c* is the cost (*c* = 0.01, 0.02,…, 0.50). The denser graphs with the cost of more than 0.50 are indistinguishable between the groups and even from the theoretical models (random and lattice), thus we did not include the range over 0.50 in our study. One might note that the selected range of cost is slightly wider than in some previous studies: 0.05 ≤ *c* ≤ 0.40 (Bernhardt et al., 2011), 0.06 ≤ *c* ≤ 0.40 (He et al., 2008), but narrower than in another study: 0 < *c* < 1 (He et al., 2009). However, determining a threshold for binary graph analysis is no trivial issue, and even selecting multiple thresholds also introduces empirical choices (Langer et al., 2013). It should be noted that using too high threshold (i.e., low cost) has a risk of excluding true connections (false negative) and too low threshold (i.e., high cost) has a risk of including false connections (false positive).

### 2.3. Graph measures: efficiency at network and node levels

In order to characterize the properties of cortical thickness networks, we used *efficiency* in this paper, which measures how efficiently a network exchanges information (Latora and Marchiori, 2001). The efficiency measure is given in two ways: (Latora and Marchiori, 2001): *global efficiency* and *local efficiency*, which are closely related to the *small-worldness* measures such as *characteristic path length* and *clustering coefficients* (Watts and Strogatz, 1998). In contrast to the small-worldness measures are defined only in a network with only one connected component, the efficiency measures are more adoptable for the real-world networks as they are also applicable to disconnected networks.

In addition to the originally proposed network-wise measures for efficiency (Latora and Marchiori, 2001), a node-wise measure has been used in the previous brain connectivity literature (Achard and Bullmore, 2007; He et al., 2009; Wang et al., 2009b; Lo et al., 2010), but only limited to the global efficiency. Combining two levels (network- and node-) and two efficiency measures (global and local), we used four different types of efficiency measures in the paper, as explained in the following subsections.

For the assessment of real-world networks from human brains, we generated cost-matched theoretical networks. The theoretical networks provide benchmarks for a network with a maximal global efficiency [i.e., a random network for unweighted graphs; Latora and Marchiori (2003)] or a network with a high local efficiency (i.e., a regular lattice) under a given constraint of cost. A “economic behavior” of network, or small-worldness, is often used to describe a network with a low characteristic path length, or a high global efficiency, as a random network and a high clustering coefficient, or a high local efficiency, as a regular lattice (Latora and Marchiori, 2003). For each level of cost, 1000 random networks with uniform probability of connections and 1000 lattice networks with the fixed patterns of adjacent connections were synthesized, then the graph measures were averaged over instances. The efficiency measures in the followings were computed using a custom modification of the MATLAB code in Brain Connectivity Toolbox^3^.

#### 2.3.1. Global efficiency

*Global efficiency* of a graph 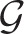 is given (Latora and Marchiori, 2001) as


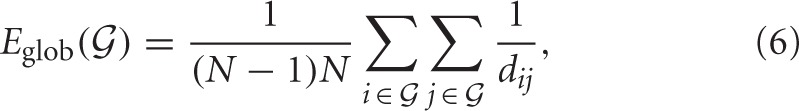


where *N* is the number of nodes in the graph 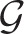, the nodes *i* and *j* within 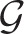 are different, (*i* ≠ *j*) and *d*_*ij*_ is the shortest path length, or geodesic distance (Newman, 2003), between the two nodes. One may note that 
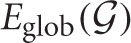
 quantifies the expectation on how closely a node is connected to all the other nodes in the whole network. It has a clear relation to a graph theoretical measure previously known as *characteristic path length* (Watts and Strogatz, 1998). While the characteristic path length is the arithmetic mean of the shortest path lengths, the *E*_glob_ is the reciprocal of the harmonic mean of the shortest path lengths (Latora and Marchiori, 2003). The *E*_glob_ is bounded from 0 to 1. When there are no connections between any nodes, all geodesic distances are equal to infinity then the *E*_glob_ equals to zero. On the other hand, when the all nodes are directly connected, all distances are equal to one and the *E*_glob_ also equals to one.

#### 2.3.2. Local efficiency

*Local efficiency* of a network 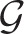 is given as the average of the global efficiencies of sub-graphs (Latora and Marchiori, 2001) as


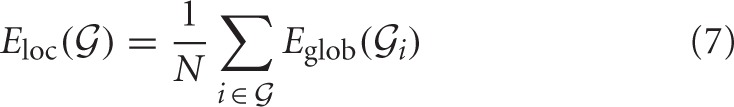


where 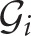 is a sub-graph centering the node *i*, that is, the set of the node *i* and its neighbors (the nodes with the distance of a single edge from the node *i*) and the set of edges between the nodes. As the measure depicts connectivity within local neighbors, Latora and Marchiori (2003) have shown that the 
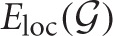
 is related to *clustering coefficient* (Watts and Strogatz, 1998). As the 
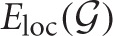
 is the average of 
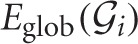
, the measure is also bounded from 0 to 1. The higher the measure, the more efficiently the nodes within a local network are interconnected.

#### 2.3.3. Nodal efficiency

Besides of network-level, we can measure how efficiently an individual node transfer information at node-level as


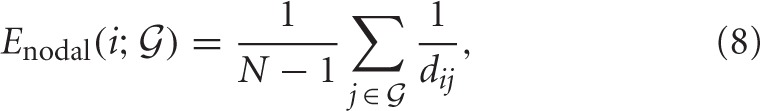


where *j* ≠ *i*. 
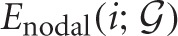
 is known as *nodal efficiency* (Achard and Bullmore, 2007). When the global efficiency 
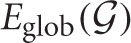
 can be understood as “the global efficiency of a network”, we can regard the nodal efficiency 
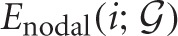
 as “the global efficiency of a node”. Remind that the term “global” or “local” only indicates whether the efficiency measure is computed for the inter-connections to the all nodes, i.e., global network, or whether it is for the intra-connections within the neighboring nodes, i.e., local network (Latora and Marchiori, 2003).

#### 2.3.4. Neighboring efficiency

The efficiency within the local neighbors of a node is computed as


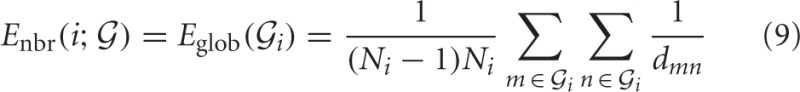


where *N*_*i*_ is the number of nodes in a sub-graph 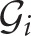 and the nodes *m* and *n* in 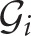 are different (*m* ≠ *n*). We call this measure 
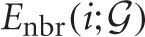
 as *neighboring efficiency*, which is a node-level measure of local efficiency, as well as the nodal efficiency 
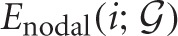
 is a node-level measure of global efficiency. By definition, 
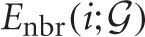
 is given when *N*_*i*_ ≥ 2, otherwise 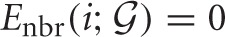 for a node with no connections or only one connection.

### 2.4. Statistical inferences

We tested the equalities of the expected efficiency measures between the controls and the OCD patients. The null hypotheses of the equality of the expected network-level efficiencies (*E*_glob_, *E*_loc_) between the networks of the controls (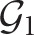) and that of the OCD patients (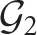) are given as


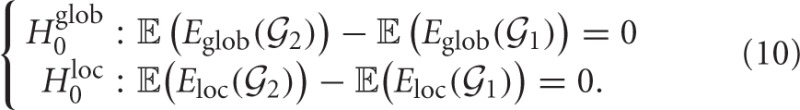


For the node-level measures (*E*_nodal_, *E*_nbr_), the null hypotheses of equality at a node *i* are given as


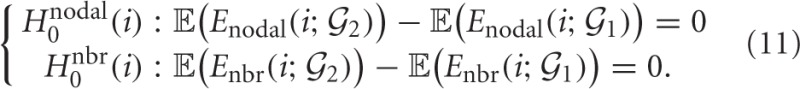


We used randomization to compute the exact *p*-values for the significances of differences (Nichols and Holmes, 2001) in the global, local, nodal and neighboring efficiency as given in section 2.3. The group identifiers (*g* = 1,2) were randomly permuted for 2000 times and the identical analysis steps were applied to construct graphs and derive efficiency measures. The difference between the two randomly separated groups were used to obtain the null distribution under the hypothesis *H*_0_ that there is no difference between the controls and the OCD patients. The *p*-values were calculated at each cost as two-tailed *p*-values.

The significance level is given as 0.05 in this study. We did not apply any multiple comparison corrections in comparing the efficiency measures for each ROI as in the previous brain connectivity studies (Achard and Bullmore, 2007; Wang et al., 2009a), neither for each cost since the networks with the adjacent costs are obviously not independent. The similar relationship between the networks across the costs has been explained by *graph filtration* (Lee et al., 2011), the process in which the succeeding network embeds the preceding network with the decreasing threshold of correlation (or the increasing “epsilon” distance in constructing *Rips complexes* (Ghrist, 2007) in an *N*-dimensional similarity space). We pursued, however, a persistent group difference over the various costs as well in this study.

## 3. Results

### 3.1. Demographic and clinical variables of participants

Demographic and clinical variables are tabulated with corresponding statistics and *p*-values in Table 1. There were no significant differences in age (*p* = 0.49), gender ratio (*p* = 0.88), education year (*p* = 0.59) and IQ (*p* = 0.49). BDI and BAI scores in the OCD patients were significantly higher than the controls (BDI, *p* < 10^−7^; BAI, *p* < 10^−6^). The mean of total Y-BOCS in the OCD patients was 21.03 with the standard deviation of 6.06.

**Table 1:** **The summary of demographic and clinical variables**.

**Variable**	**Controls (*n* = 35)**	**OCD patients (*n* = 32)**	***t*-/*z*-stat.**	***p*-value**
Age (year)	23.94 ± 3.60	24.81 ± 6.41	0.69	0.49
Gender (men/women)	24/11	21/11	0.15	0.88
Education (year)	14.03 ± 1.29	14.34 ± 3.23	0.53	0.59
IQ	113.20 ± 9.98	111.40 ± 11.32	−0.69	0.49
BDI	4.26 ± 6.17	17.37 ± 10.71	6.21	<10^-7^
BAI	4.54 ± 5.49	18.72 ± 13.70	5.65	<10^-6^
Y-BOCS				
Obsession	–	11.97 ± 3.49	–	–
Compulsion	–	9.06 ± 4.69	–	–
Total	–	21.03 ± 6.06	–	–

*The mean and the standard deviation of the variables are shown except for gender. The IQ was estimated by Korean-Wechsler adult intelligence scale-revised (K-WAIS-R)*.

Abbreviations *BDI* *Beck's depression inventory* *BAI* *Beck's anxiety inventory* *Y-BOCS* *Yale-Brown obsessive-compulsive disorder Scale*.

Out of 32 OCD patients, 13 patients (41%) were with contamination, 8 patients (25%) with checking, 5 patients (16%) with aggressions and 5 patients (16%) with other obsessions of sex, religion, somatic, or a combination of them as their prominent symptoms, as classified with Y-BOCS Symptom Checklist (Goodman et al., 1989). The main symptom of one patient was not determined. No significant differences between the two largest subgroups (contamination *vs.* checking) were found in total Y-BOCS (*p* = 0.31), neither in obsession (*p* = 0.10) nor compulsion (*p* = 0.98) subscores. The equalities across the other subgroups were not tested due to the small sizes of the subgroups. In addition, we did not find the effect of the history of medications on the severity of OCD either; between 23 drug-naïve patients and 8 unmedicated patients, there were no significant differences in total Y-BOCS (*p* = 0.25) and the subscores of obsession (*p* = 0.90) and compulsion (*p* = 0.58).

### 3.2. No group differences in cortical thickness and correlation coefficients

In prior to graph measure analysis, we compared cortical thickness covayring age and gender with multiple comparison correction by SurfStat MATLAB toolbox^4^ (Worsley et al., 2009). We found no significant group differences (Figure not shown; corrected *p* > 0.56 in left hemisphere and *p* > 0.16 in right hemisphere). In addition, inter-regional correlations *r*_xy_ as given in (Equation 2) were compared between groups using Fisher's *z*-transformation. Due to the substantially large number (148 × 147/2 for all possible pairs) of simultaneous testings, false-discovery-rate (FDR; Benjamini and Hochberg, 1995) is used for this case. Once again, no correlation between the pairs of nodes were found to be significantly different between the patients with OCD and the controls (*q* > 0.40).

### 3.3. Small-worldness of the brain networks

**Figure 2:**
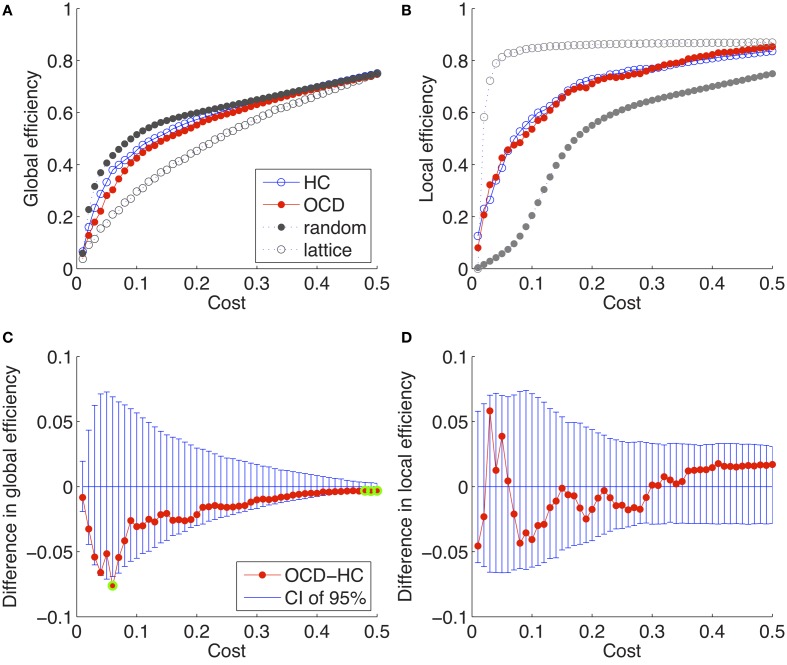
**Global efficiencies and local efficiencies of the brain networks of the controls (HC), and the OCD patients (OCD) are plotted over variant rewiring costs with the ones of random and lattice networks with matched costs (**A**,**B**).** The differences between the OCD patients and controls are plotted with the 95% confidence intervals (CI) of the null distributions obtained from 2000 permutations **(C,D)**. The significantly different points (*P* < 0.05) are marked with green circles **(C)**. Note that no significant differences were found in the local efficiencies.

The global efficiencies and local efficiencies of the brain networks, as well as the random and lattice networks with the matched costs, are given over the varying costs (0.01, 0.02, …, 0.50) in Figure 2. We found that the network-level efficiency measures of the brain networks were invariently in-between the cost-matched random and lattice networks as


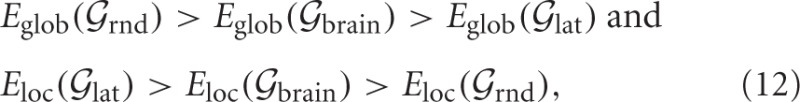


except for two extreme cases (cost of 0.01 and 0.50).

These characteristics of inequalities have been typically referred as economic small-world behaviors of networks (Latora and Marchiori, 2003; Achard and Bullmore, 2007). It has been found that many brain networks of clinical populations are still in the small-world regime despite the significantly altered properties of patients in comparison with the healthy populations (He et al., 2007, 2008, 2009; Wang et al., 2009b; Lo et al., 2010). Thus we presume that the cortical thickness network of the OCD patients has the small-world architecture, as well as that of the controls.

### 3.4. No group differences in network-level efficiency

We found significantly smaller global efficiencies in the OCD patients than the controls at the cost of 0.06 (*p* = 0.03), 0.48 (*p* = 0.04) and 0.49 (*p* = 0.02), but found no differences at the other costs. No significant group differences in local efficiency were found at any costs we studied. In addition, the area under curves (AUC) divided by the range of costs, or the mean of efficiencies across the discrete costs, were compared. We found no differences in the mean global efficiency (*p* = 0.14) nor the mean local efficiency (*p* = 0.74). Taken together, we did not find a clear distinction between the OCD patients and the controls in terms of the aggregated network-level efficiency measures.

### 3.5. Group differences in node-level efficiency

**Figure 3:**
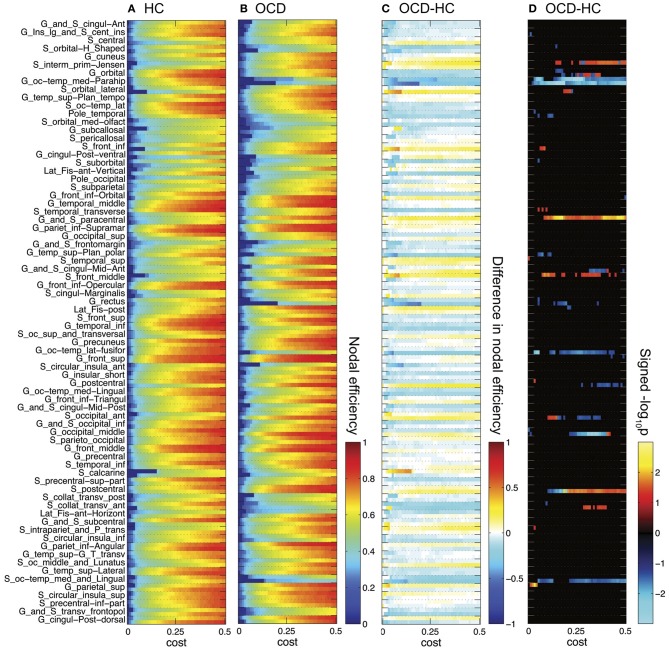
**Nodal efficiencies for 148 ROIs are plotted over cost for the controls (HC, **A**) and the OCD patients (OCD, **B**).** Along the Y-axis, 74 ROI labels are indicated for each set of two rows separated by dashed horizontal lines (upper row for the left hemisphere; lower row for the right hemisphere). See Destrieux et al. (2010) for abbreviation of the ROI labels. The group differences as HC subtracted from OCD are shown **(C)**. The warm color shows higher nodal efficiency in OCD than in HC, and the cool color shows the opposite. The signed logarithmic *p*-values for the significance of group differences are also given **(D)**. The positive values means higher nodal efficiency in OCD than in HC (−log_10_ *p* > 0), and vice versa (log_10_*p* < 0).

**Figure 4:**
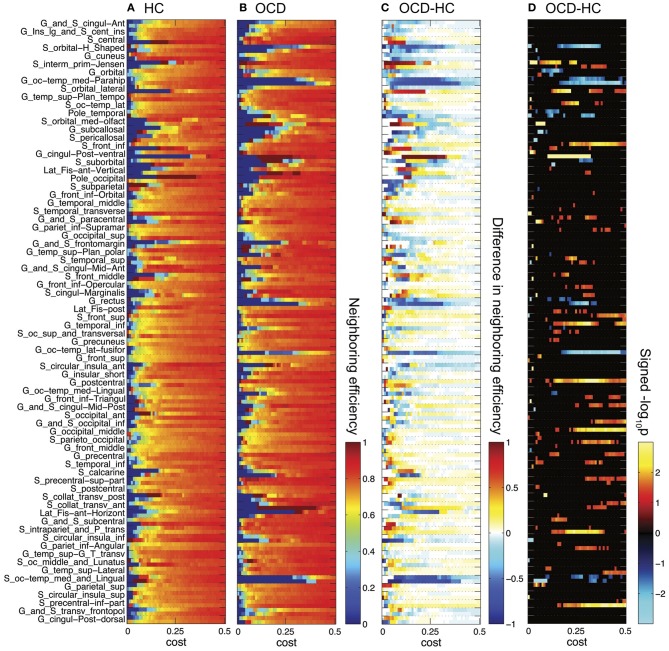
**Neighboring efficiencies for 148 ROIs are plotted over cost for the controls (HC, **A**) and the OCD patients (OCD, **B**).** The group differences as HC subtracted from OCD **(C)** and the signed logarithmic *p*-values **(D)** are also shown. The same graphical scheme is the same as Figure 3. The warm color means higher neighboring efficiency in OCD than in HC and the cold color means the opposite **(C,D)**.

In contrast to the results of network-level efficiency, we found significant group differences at node-level. The heat maps of group-wise nodal efficiency and neighboring efficiency over costs are given in Figures 3, 4, respectively. Additionally, the differences in efficiency measures between the groups and the negative logarithm with the base of ten of *p*-values are also shown together. The logarithmic *p*-value is signed as positive when the efficiency is greater in the OCD patients than in the controls (−log_10_ *p* > 0) and as negative when the value is smaller in the patients than in the controls (log_10_ *p* < 0).

What can be prominently noted from Figure 3 is that the number of disconnected nodes (the nodes with infinity distance to all other nodes thus zero nodal efficiency; blue pixels in Figures 3A,B) at a low cost are larger in the OCD patients than in the controls (e.g., when *c* = 0.01, 74 in OCD, 61 in HC), and that it takes higher costs to be connected to any nodes in the OCD patients (*c* = 0.20) than in NC (*c* = 0.15). Unlike the small-wordness measure (Watts and Strogatz, 1998), which is given for a connected network, the efficiency measure (Latora and Marchiori, 2003) enables the investigation of the disconnected graphs with smaller costs in the present study.

As we have done previously for the network-level efficiency measures, the AUC divided by the range of costs were compared for each node, so that we can compare mean measures across costs. Out of the 148 ROIs, 9 nodes showed significant group differences in nodal efficiencies, while 15 nodes were found to be significantly different in neighboring efficiencies (*p* < 0.05), as summarized in Table 2. For frontal regions, left orbital frontal gyrus and right lateral orbital sulcus showed lower neighboring efficiencies in the OCD patients than the controls, while left middle frontal sulcus exhibited smaller nodal efficiency as well. On the other hand, parietal regions such as left postcentral gyrus, right postcentral sulcus, left superior parietal gyrus, and bilateral sulci intermedius primus of Jensen showed higher efficiency measures in the OCD patients. Finally, medial occipito-temporal gyri around parahippocampal gyri bilaterally showed smaller nodal and neighboring efficiencies in the patients with OCD, with smaller nodal efficiency in right lateral and medial occipito-temporal gyri.

**Table 2:** **The summary of the nodes with significant differences in mean efficiency (*p* < 0.05) as the subtraction of controls (HC) from the OCD patients (OCD-HC) with corresponding *p*-values for nodal efficiency (A) and neighboring efficiency (B)**.

**Location**	**Structure name**	**OCD-HC**	***p*-value**
**A. Nodal efficiency (*E*_nodal_)**			
Dorsal nodes	Paracentral loblue and sulcus	0.1699	0.023
	Left middle frontal sulcus	0.2002	0.037
	Right postcentral sulcus	0.1316	0.034
	Right middle occipital gyrus	0.0944	0.041
Ventral nodes	Left parahippocampal part of the medial occipito-temporal gyrus	−0.2836	0.015
	Right parahippocampal part of the medial occipito-temporal gyrus	−0.3653	0.001
	Right gyrus rectus	−0.2620	0.049
	Right lateral occipito-temporal gyrus	−0.2427	0.034
	Right medial occipito-temporal sulcus and lingual sulcus	−0.2690	0.026
**B. Neighboring efficiency (*E*_nbr_)**			
Dorsal nodes	Left postcentral gyrus	0.1208	0.019
	Left superior parietal gyrus	0.0989	0.033
	Left sulcus intermedius primus of Jensen	0.3367	0.014
	Left superior part of the precentral sulcus	0.1552	0.027
	Right sulcus intermedius primus of Jensen	0.1787	0.030
	Right intraparietal sulcus and transverse-parietal sulcus	0.1349	0.024
Ventral nodes	Left H-shaped orbital sulcus	−0.4093	0.004
	Left orbital gyrus	−0.1367	0.032
	Left parahippocampal part of the medial occipito-temporal gyrus	−0.4367	0.015
	Left inferior temporal gyrus	0.0722	0.047
	Left medial occipito-temporal sulcus and lingual sulcus	−0.4374	0.035
	Right parahippocampal part of the medial occipito-temporal gyrus	−0.5707	0.003
	Right lateral orbital sulcus	0.3416	0.028
	Right lateral occipito-temporal gyrus	−0.3742	0.002
	Right horizontal ramus of the anterior segment of the lateral fissure	−0.5793	0.011

*Dorsal nodes and ventral nodes are separated for tabulation*.

#### 3.5.1. Spatial pattern of node-level efficiency differences

**Figure 5:**
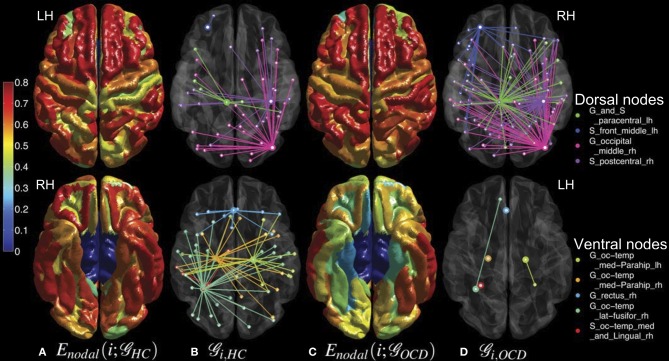
**Mean nodal efficiencies across costs are overlaid on the cortical surfaces of the controls (HC, column **A**) and the OCD patients (OCD, column **C**) from a dorsal view (upper row) and a ventral view (lower row).** See the color bar on the leftmost side for the color coding from 0 to 0.8. The sub-graphs (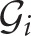) of the binary networks at the cost of 0.10 are shown as well for HC (column **B**) and OCD (column **D**). From the nodes that showed significantly different nodal efficiencies (marked by thick circles), their first neighbors are connected in distinct colors which correspond to the ROI legend on the rightmost side.

**Figure 6:**
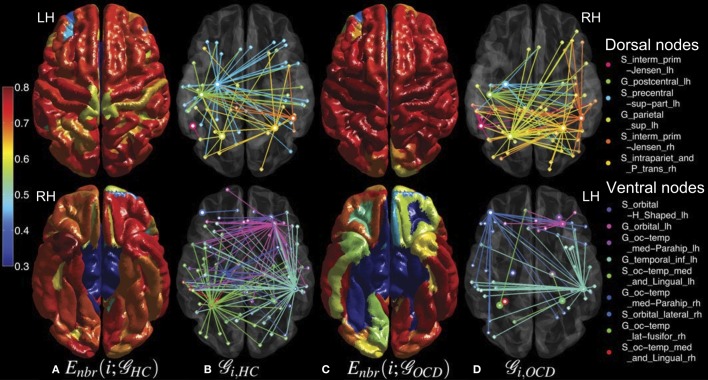
**Mean neighboring efficiencies across costs for the controls (HC, column **A**) and the patients with OCD (OCD, column **C**) and the sub-graphs** (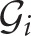) **of the corresponding binary networks at the cost of 0.1 (HC, column B; OCD, column D) are shown in the same graphical scheme as Figure 5, except that the color coding range of neighboring efficiencies is adjusted from 0.3 to 0.8 for a better visualization**.

**Figure 7:**
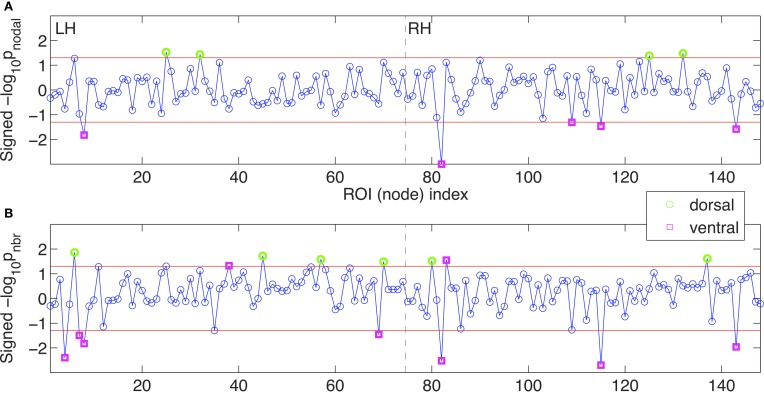
**The mean node-level efficiency measures were compared.** Signed logarithmic *p*-values for nodal efficiency **(A)** and for neighboring efficiency **(B)** are given. The x-axis indicates the index of the ROI, or the node, from the left hemisphere (LH) to the right hemisphere (RH), which are separated by dashed vertical lines. Along the y-axis, the positive values mean the higher efficiency measures in OCD patients than in controls (-log_10_*p* > 0), and the negative values mean lower efficiency measures in OCD patients (log_10_*p* < 0). The significance level of α = 0.05 are marked with red horizontal lines and the suprathreshold nodes are highlighted by green circles (dorsal nodes) or magenta squares (ventral nodes). See Table 2 for ROI labels.

Interestingly, the spatial bias of the node-level differences in efficiency measures was found at a large scale (i.e., dorsal *vs.* ventral). For the sake of simplicity, the nodes are classified either as a dorsal or ventral node, based on the Z-coordinate of the center of mass of a ROI, in relation to the median of Z-coordinates of the all ROIs. Mind that this separation based on the Z-coordinate is only for the purpose of a simple comparison of the spatial distribution. Further investigation on community structures based on thickness correlation also might be useful to analyze the distribution of local alterations, but we did not include such an analysis in the present study to keep our focus here to the efficiency of networks. The geometrical distribution of the efficiency measures and the corresponding binary networks in the template space are visualized in Figures 5, 6, for nodal efficiency and neighboring efficiency, respectively. As previously hinted at by Table 2, it can be noticed that the efficiency measures of many dorsal nodes are greater in the OCD patients than the controls, and the ones of many ventral nodes are smaller from Figures 5, 6. The signed *p*-values of 148 ROIs are summarized while the location (dorsal or ventral) of nodes with significant group differences marked in Figure 7. For the nodal efficiency *E*_nodal_ (Figure 7A), all of the 4 nodes with significantly larger values in the OCD patients (nodes above the upper red line) were dorsal without any ventral nodes (100%; green), and all of the 5 nodes with significantly smaller values (nodes under the lower red line) were ventral (100%; magenta). For the neighboring efficiency *E*_nbr_ (Figure 7B), 6 out of 8 nodes with significantly greater values in the OCD patients were dorsal (75%), and all of the 7 nodes with significantly smaller values were ventral (100%).

Although the spatial bias in the present results seems clearly discernible (100%; 100%; 75%; 100%), one may be interested in the stability of the present finding. It can be possible to compute reliability using a resampling method known as jack-knifing. Unfortunately, however, we used randomization to compute *p*-values for group differences. To see the spatial pattern of the nodes with significantly different efficiency in a resampled subset, each subset requires a new run of randomization and graph measure computation. It renders impractical computational load with the current MATLAB codes. Thus we have not carried out the analysis for this study. We discuss on the spatial bias of node-level efficiency measures between the dorsal and ventral networks from the perspective of the imbalance theory of dorsal-ventral pathways in the OCD patients in the following section.

#### 3.5.2. Altered relationship between node-level efficiency and node centrality

**Figure 8:**
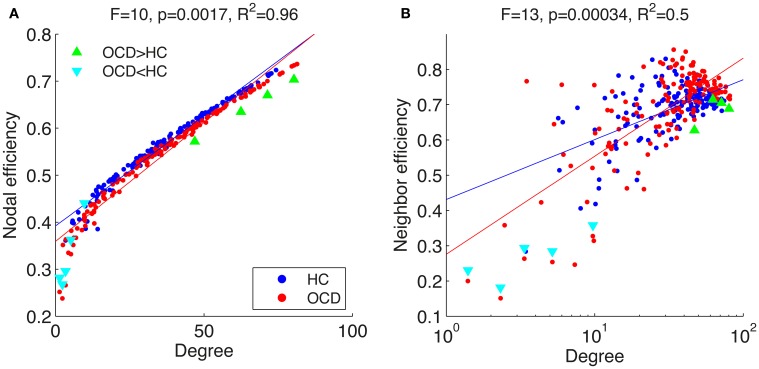
**Mean nodal efficiency (**A**) and neighboring efficiency (**B**) are shown over mean degrees in a linear (**A**) and a logarithmic (**B**) scale.** Each point indicate an ROI from the networks of the healthy controls (HC, blue dots) and patients with OCD (OCD, red dots). Specific ROIs with significant group differences in efficiency measures are marked with either green triangles (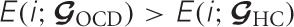) or cyan triangles (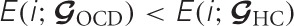). Regression lines are given for each group (HC, blue line; OCD, red line). Above panels, *F*-statistic and *p*-value for a GLM testing the interaction between logarithm of degree and group and *R*^2^ for the full model are given.

In addition to the spatial pattern, the relationship between the efficiency and centrality are further examined. We used degree, which is the number of connected edges to a node, as a simple measure for node centrality. The mean efficiencies are plotted over degrees in Figure 8. The efficiency is fitted using a GLM as

(13) $$E(i)=\beta_{0}+\beta_{1}D(i)+\beta_{2}G(i)+\beta_{3}D(i)G(i)+\varepsilon$$

where *E*(*i*) is an efficiency measure of the *i*-th node, *D*(*i*) is a degree and *G*(*i*) is a group index. The efficiencies were well explained by the full models (nodal efficiency, *R*^2^ = 0.98; neighboring efficiency, *R*^2^ = 0.50). We used logarithm of degrees when the model fit is improved as in case of neighboring efficiency (*R*^2^ for the full model with a linear degree measure is 0.39).

**Figure 9:**
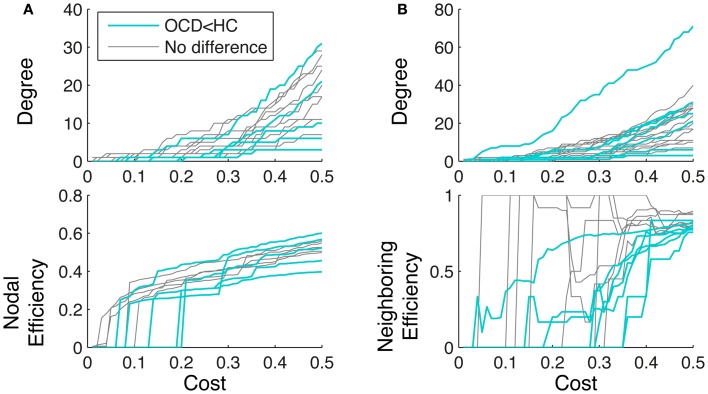
**Degree (upper row) and efficiency (lower row) are plotted over cost for the nodes with smaller nodal efficiency (**A**, cyan) and smaller neighboring efficiency (**B**, cyan) from OCD patients.** For comparison, the measures of the other nodes from the patients with similar mean degrees are also shown (gray). The nodes with smaller efficiency in OCD than healthy controls exhibits different growth of efficiency even though the mean degrees are similar to the other nodes in OCD.

The interactions between group index and degree were found to be significant for nodal efficiency (*p* < 0.005) and neighboring efficiency (*p* < 0.0005). The nodes with significantly difference efficiencies seem to be responsible for the interaction, especially for that the nodes with lower efficiency are deviated from the fitting lines. The *p*-values for the interactions without the nodes with significantly smaller efficiencies were higher than significance level in the study (nodal efficiency, *p* = 0.23; neighboring efficiency, *p* = 0.24). Thus the nodes with significantly smaller efficiencies in patients with OCD seem to be aberrant from the other nodes in the OCD patients. As an illustration for this idea, the degrees and efficiencies of the nodes with the smaller efficiencies from OCD patients are plotted over cost in Figure 9. For comparison, the measures of the other nodes with similar degrees from the patients are also given. In the process of graph growth with the increasing cost, the efficiencies of the nodes without significant group differences (gray lines) increase earlier than the nodes with significant group differences (cyan lines). Thus the nodes with group differences have smaller mean efficiency (AUC divided by the range of cost) than the other nodes with similar mean degrees, deviating from the fitting lines in Figure 8.

## 4. Discussion

### 4.1. The brain network of OCD patients in the small-world regime

We found that the brain network of the OCD patients is within the small-world regime as well as that of the controls. The small-worldness of neuronal network has been demonstrated in various scales: the neuronal system of *C. elegans* (Watts and Strogatz, 1998), the brains of cats and macaque monkeys (Hilgetag and Kaiser, 2004; Kaiser, 2007), and that of humans (Sporns et al., 2004; Sporns and Honey, 2006; Achard and Bullmore, 2007; Hagmann et al., 2007; He et al., 2007; Hagmann et al., 2008). Although the network properties were found to be altered to a significant degree, the brain networks of clinical population also exhibited small-worldness distinctively from the cost-matched theoretical networks (He et al., 2008, 2009; Wang et al., 2009a; Bernhardt et al., 2011). In consistence with a previous functional network study on the OCD patients (Zhang et al., 2011), we confirmed that the structural network of the patients shows small-worldness as well in terms of inequality of network-level efficiency measures (Equation 12) as shown in Figure 2.

**Figure 10:**
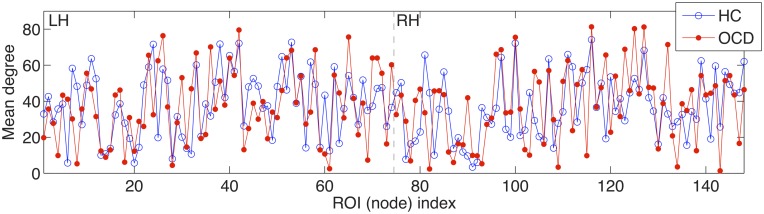
**The averaged degrees across costs over 148 ROIs are shown for the controls (HC) and OCD patients (OCD) on the y-axis.** Similarly to Figure 7, the x-axis indicates the index of the ROI from the left hemisphere (LH) to the right hemisphere (RH), which are separated by dashed vertical lines.

The small-worldness of a network implies the existence of local clusters in relation to its equivalent random counterpart (Kaiser, 2011). As it can be seen in Figures 1E, 10, the cortical thickness networks from both of the OCD patients and the controls remarkably showed the variant degrees of connections across nodes. Regarding that the random and lattice networks are generated to have uniform distributions of degrees, the variety of degrees of the real-world brain networks makes them clearly distinguishable from the theoretical networks. In particular, the pattern of mean degrees showed noteworthy resemblance between the brain networks of the OCD patients and the control as given in Figure 10. The correlation of mean degrees between the groups was strongly positive (*r* = 0.4969, *p* < 10^−9^). It may imply that the essential structures and functions of brain network are still preserved in the OCD patients, as shown as intact capabilities of basic behaviors and primitive functioning of the patients, though diverse impairment in high level cognitive functioning (Graybiel and Rauch, 2000; Kuelz et al., 2004).

### 4.2. Dorsal and ventral disparity in OCD patients

The most significant contribution of our present study is detecting the disparity between the dorsal and the ventral networks in the OCD patients in terms of graph theoretical measures, supporting the hypothesis on “the imbalance of tone” between direct and indirect CST pathways (Saxena et al., 1998, 2001).

Although we could not find any group differences in the mean network-level efficiency measures, we found significant alterations of the node-level efficiency in the OCD patients that were localized with an evident spatial bias as shown in Figures 5, 6, which may reflect the imbalance between the dorsal and ventral pathways in the patients. In particular, the topological alterations were particularly localized in sensory-motor regions including paracentral lobule, postcentral regions, parietal cortices and middle frontal cortex as greater nodal or neighboring efficiencies in the OCD patients than the controls, and the aberrations were also detected in the ventral frontal and temporal regions including orbital cortices the parahippocampal cortices as smaller nodal or neighboring efficiencies in the OCD patients. We have shown that the ratios of nodes with significantly greater or smaller efficiency are not equal between the dorsal and ventral nodes, which may reflect the spatial disparity of the subnetworks in the patients with OCD, as shown in Figure 7.

Our findings are in accordance with the previous VBM studies those found local alterations of gray matter in parietal cortex (Kim et al., 2001; Valente et al., 2005), middle temporal and occipital cortex (Togao et al., 2010) and orbital frontal cortex (Pujol et al., 2004; Szeszko et al., 2008). Given the neuropathological model (Saxena et al., 2001; Menzies et al., 2008), those regions have been considered as the loci of the abnormalities of OC symptoms such as attention control deficit, excessive anxiety and failure of impulse control (Friedlander and Desrocher, 2006; Menzies et al., 2008).

More interestingly, the present findings seem to be closely related to a multivariate study on the structural network of OCD patients (Menzies et al., 2007), which used a statistical technique called partial least square (PLS; McIntosh et al., 1996). Unlike the massive univariate approaches such as VBM, PLS extracts spatial patterns that optimally correlate with a given measure of interest from the whole image. Thus the ability of PLS to detect a component with covariance is quite similar to that of the cortical thickness network analysis we used in this paper, in the sense of multivariate approaches. Their study showed that higher gray matter density in a “parieto-cingulo-striatal system” and lower gray matter density in a “fronto-temporal system” were correlated with increasing behavioral impairment in OCD patients (Menzies et al., 2007), with a striking congruence with the current findings.

### 4.3. Aberrant relationship between efficiency and centrality in OCD patients

We also found that significant interactions between degree centrality and group on node-level efficiency. The interactions seemed to be driven by the nodes that showed significant group differences, which are deviated even from the other nodes within the patients. In particular, the nodes with smaller efficiency in OCD patients demonstrated different graph growth trajectory with the increasing costs, compared to the other nodes in OCD patients with similar mean degrees. The nodal efficiency of a node can increase without the additional connections to the node but by the additional connections to the other node that is already connected to the node. In other words, a node that is connected to a hub node can have high nodal efficiency even with only 1°. In case of OCD patients in the present study, the nodes without group differences (Figure 9A, gray lines) showed abrupt increase of nodal efficiency at a low cost (<0.05) without much increase of degrees. On the other hand, the nodes with group differences (Figure 9A, cyan lines) took high costs to have sudden increase of nodal efficiency, which is likely to be the point when the node is connected to the other node with high degrees.

Neighboring efficiency does not monotonously increase by increasing cost, since the measure quantifies the connections between the first neighbors of the node. Thus the neighboring efficiency can suddenly decrease during the graph growth when a new node without any connections to the pre-existing first neighbors is connected. In our case of OCD patients, the nodes without group differences (Figure 9B, gray lines) showed sudden increases at low degrees and sudden decrease, while the nodes with group differences (Figure 9B, cyan lines) evolved with slowly increasing neighboring efficiency, which means connecting unrelated nodes into its the first neighborhood.

Only 3 nodes out of 24 nodes with group difference in efficiency found to be with significantly different degree between groups (*p* < 0.05). It is presumably because that the nodes with significant group differences showed different trajectory of evolution. Thus the difference in node level efficiency could not be explained solely by degree centrality but also the connectivity of other nodes as well. As an alternative measure for centrality in a close relation with efficiency, *information centrality* has been introduced (Latora and Marchiori, 2004), but further investigation in terms of graph theories remains to pursuit in this paper.

### 4.4. Cortical thickness network and the pattern of functional activations in OCD patients

The disruption of structural architecture may correlate with the alteration of the functional activation of involved areas. Specifically, the cortical thickness network has demonstrated its spatial correspondence to the DMN in human brains (Raznahan et al., 2011), and the altered relationship between the subcortico-cortical structures in a mouse model of Huntington's disease, in which its subcortical functions were impaired by a gene-knockout (Lerch et al., 2008). Here, the altered efficiency measures at a node level in the cortical thickness network of OCD patients implicate the different pattern of one-to-*n* similarity of local morphology of a node (nodal efficiency), or the different pattern of *n*-to-*n* similarity within the neighbors of the node (neighboring efficiency) in relation to the network of the controls. Although we do not have concurrent functional dataset of the participants, an fMRI meta-analysis using activation likelihood estimation (ALE; Turkeltaub et al., 2002) demonstrated that a greater activation was found in the left inferior parietal cortex in OCD patients than controls and a smaller activation in the left parahippocampal gyrus was found during various tasks (Menzies et al., 2008) in relation to our current findings. However, it would be fair to note that the meta-analysis also reported the foci of abnormal activations that are not clearly relevant to our results, as well as other studies that showed the different patterns of activation in OCD patients, during tasks (Nakao et al., 2005; Han et al., 2011) and using PET at rest (Kwon et al., 2003). Thus the relationship between the patterns of functional activation and the efficiencies of cortical thickness network may not be simply straightforward but manifold due to the complex nature of human brains.

A recent whole-brain analysis on the functional connectivity of OCD patients showed significantly higher or lower inter-regional correlations of activations at rest (Zhang et al., 2011). The spatial patterns of aberrant functional connectivity in their findings were not quantified, but it can be noted that higher correlations were found between parietal nodes, cingulate nodes and dorsal frontal nodes and lower correlations were found between prefrontal nodes and posterior temporal nodes (Zhang et al., 2011). Although a larger number of samples and simultaneous multi-modal data will be beneficial to clarify the interaction between the structural and functional networks, we conjecture that the topological alteration in the structural network in OCD patients would exhibit concurrent deviant patterns of functional activations.

It can be added to discussion, that the resting-state hyperactivity in the ventral networks in OCD patients has been consistently found in terms of greater local activation (Kwon et al., 2003; Friedlander and Desrocher, 2006) and higher cortico-strial functional connectivity of the basal ganglia (Harrison et al., 2009). Intriguingly, it was demonstrated that the ventral network, primarily including orbital frontal cortex, showed smaller deactivation (i.e., the failure of inhibition), responding to the participant's own error, thus resulting in higher activation in OCD patients compared to controls (Stern et al., 2011). The coarse connections and lower efficiency in the ventral network of the OCD patients in our present findings implies that the morphometric similarity of the ventral nodes with other nodes is disturbed. It may reflect the underlying pathology of the dysfunction of inhibitory controls in the OCD patients.

### 4.5. Limitations and future works

The first methodological limitation of our study is that the current practice of cortical thickness network analysis is restricted to cortical structures. Although the subcortical structures were considered to be highly involved in the pathophysiology of mental disorders including OCD (Cummings, 1993; Saxena et al., 2001), the present technical issues such as MR imaging resolution and tissue contrast still render the surface analysis problematic to the other brain structures than neocortices, despite recent computational advances (Khan et al., 2008; Qiu et al., 2010). Alternatively, the volumetric measure of a subcortical structure may be used along with the cortical thickness (Lerch et al., 2008). In addition, it can also be possible to characterize the covariance structure of local morphology in volumetric space (Kim et al., 2011a; Tijms et al., 2012). It may be useful to adapt and combine those methods to investigate the relationship within and between the cortical and subcortical networks.

The second limitation is that we could not separate the OCD patients by their main symptoms, mainly due to the small size of subgroups. As there have been rich discussions and supporting evidences for the heterogeneity of OCD symptoms (Mataix-Cols and van den Heuvel, 2006; van den Heuvel et al., 2009; Koch et al., 2012), possible subtypes and multi-dimensions of the disorder were discussed in the context of refining the diagnosis criteria in the next generation of DSM (Leckman et al., 2010; Mataix-Cols et al., 2010; Taylor, 2011). Even though we did not carry out the analyses on the subgroups of the OCD patients in this paper, a methodological improvement of the diagnosis and a larger number of samples may resort the inconsistency in the previous findings due to the diversity of OCD.

In relation to heterogeneity, we did not find any group differences in cortical thickness in the current sample. Although we previously reported cortical thinning in other patients with unmedicated OCD (Shin et al., 2007), it was demonstrated that a severity of OCD subtype may be correlated with the cortical thickness (Nakamae et al., 2012). The underlying mechanism of OCD might not be directly reflected in the local morphometry, but rather be manifested in the interaction of complex networks, which motivated the series of graph analysis on human brain including the current study as well.

In conclusion, we have examined the network properties in the patients with OCD based on the cortical thickness for the first time. The anatomical network in the OCD patients was in the small-world regime as well as that of the healthy controls. We found topological alterations in the patients in terms of efficiency at node level and its relation to node centrality. The alteration showed disparity between the dorsal and ventral networks, which may contribute to confirm the dorsal-ventral imbalance hypothesis (Saxena et al., 2001).

### Conflict of interest statement

The authors declare that the research was conducted in the absence of any commercial or financial relationships that could be construed as a potential conflict of interest.

## Footnotes

^1^http://surfer.nmr.mgh.harvard.edu

^2^http://brainimaging.waisman.wisc.edu/~chung/lb/

^3^https://sites.google.com/site/bctnet/

^4^http://www.math.mcgill.ca/keith/surfstat/
